# Frequency of Biopsy and Tumor Grade Before vs After Introduction of Prostate Magnetic Resonance Imaging

**DOI:** 10.1001/jamanetworkopen.2023.30233

**Published:** 2023-08-22

**Authors:** David Robinson, Rafid Abdulkareem, Delshad Nasrollah, Anders Ljung, Per Hintze, Sara Wallby, Henriettæ Ståhlbrandt, Thorun Frennvall, Johan Styrke, Pär Stattin, Hans Garmo

**Affiliations:** 1 Department of Urology, Highland Hospital, Eksjö, Sweden; 2 Department of Radiology, Highland Hospital, Eksjö, Sweden; 3 Department of Pathology, Ryhov County Hospital, Jönköping, Sweden; 4 Department of Surgical and Perioperative Sciences, Urology and Andrology, Umeå University, Umeå, Sweden; 5 Department of Surgical Sciences, Uppsala University, Sweden

## Abstract

**Importance:**

In randomized clinical trials (RCTs), magnetic resonance imaging (MRI) before prostate biopsy has been associated with fewer biopsies, decreased detection of Gleason score 6 cancers, and increased detection of Gleason score 7 or higher cancers.

**Objective:**

To study whether MRI of the prostate before the decision to biopsy is associated with biopsy frequency and distribution of Gleason score in clinical practice.

**Design, Setting, and Participants:**

This is a retrospective, population-based cohort study of men in Jönköping Region, Sweden. Men with prostate-specific antigen (PSA) level measured between November 2011 and 2020 were monitored until January 31, 2021. Men with known prostate cancer were excluded. Data analysis was performed from July to December 2022.

**Exposures:**

Data on repeated PSA measures, prostate biopsies, and MRI prostate were extracted from health care records, and cancer characteristics were obtained from The National Prostate Cancer Register.

**Main Outcomes and Measures:**

The proportions of men who underwent prostate biopsy and risk of Gleason score 6 or Gleason score 7 or higher cancer and negative biopsy before and after introduction of MRI were calculated.

**Results:**

In this cohort study of 23 802 men (mean [SD] age, 60.8 [13.6] years) who underwent PSA testing, when the use of MRI increased, fewer biopsies were performed (adjusted odds ratio [OR], 0.84; 95% CI, 0.72-0.97) and the odds of detecting Gleason score 6 cancer decreased (OR, 0.47; 95% CI, 0.33-0.64), whereas the odds of detecting Gleason score 7 or higher cancer increased (OR, 1.24; 95% CI, 1.02-1.50).

**Conclusions and Relevance:**

In this study, the introduction of MRI to clinical practice was associated with a decreased proportion of men who underwent a biopsy and decreased detection of Gleason score 6 cancer but increased detection of Gleason score 7 or higher cancer. These clinical data support the use of prostate MRI before biopsy in an effort to avoid unnecessary biopsies.

## Key Points

**Question:**

Is the use of magnetic resonance imaging (MRI) associated with fewer biopsies among patients with suspected prostate cancer?

**Findings:**

In this cohort study of 23 802 Swedish men, the use of MRI before a biopsy decision was associated with a lower proportion of men who underwent biopsy, detection of fewer Gleason score 6 cancers, and detection of more Gleason score 7 or higher cancers.

**Meaning:**

These data suggest that prostate MRI before biopsy may help reduce unnecessary biopsies.

## Teaser

This cohort study examines whether magnetic resonance imaging of the prostate before the decision to biopsy is associated with biopsy frequency and distribution of Gleason score in clinical practice.

## Introduction

Magnetic resonance imaging (MRI) of the prostate has changed the diagnostic workup of men with increased levels of prostate-specific antigen (PSA).^1^ National and international guidelines^2,3^ currently recommend that MRI be performed before a decision is made whether to perform a prostate biopsy. An increasingly common pathway is that when the MRI findings are abnormal, MRI-targeted biopsies are obtained, whereas men with normal findings on MRI do not undergo biopsies. In some randomized clinical trials (RCTs), this new pathway was associated with the detection of fewer Gleason score 6 cancers, whereas the detection of Gleason score 7 or higher cancers increased.^4,5^ In a recent screening study,^6^ MRI-targeted prostate biopsies compared with targeted plus systematic biopsies were associated with decreased proportions of Gleason score 6 cancers detected at the expense of a small delay in the detection of Gleason score 7 or higher cancers. However, it is unknown whether this diagnostic pathway can be reproduced in clinical practice because the case mixes in RCTs rarely mirror the case mixes seen in clinical practice, and this discrepancy may result in poor external validity of results from RCTs.^7^ The aim of this cohort study was to investigate whether the introduction to clinical practice of MRI-targeted biopsies of men with moderately elevated PSA was associated with a decrease in prostate biopsies and whether it was associated with the detection of Gleason score 6 and Gleason score 7 or higher cancer.

## Methods

We followed the Strengthening the Reporting of Observational Studies in Epidemiology (STROBE) reporting guideline for cohort studies. The Swedish Ethics Review Authority approved this study. As in all Swedish clinical cancer registers and other quality registers, all men with prostate cancer are informed at time of diagnosis and treatment that they will be included in Sweden’s National Prostate Cancer Register (NPCR) unless they opt out; therefore, written informed consent was not required.

In Sweden, health care is provided by 21 independent regions, and public health care is tax funded. According to Statistics Sweden,^8^ Jönköping Region had a male population of approximately 140 000 in 2015, which increased to approximately 150 000 in 2020. There are virtually no private health care institutions or practitioners; all laboratory testing, including biochemical analyses, pathology reports, and imaging, is provided by public health care entities, including clinics and hospitals and individual health care practitioners.

All men in Jönköping Region whose PSA was measured between November 1, 2011, and July 31, 2020, were included in this study; follow-up ended on January 31, 2021 (eFigure in Supplement 1). Men with known prostate cancer on November 1, 2015, were excluded from the study. We extracted data on prostate biopsies and prostate MRI from health care records. We then restricted the study group to men whose PSA had not been measured during the 4 years before the study period. The 4-year run-in period made men included early or late comparable because they had the same PSA measurement history. If a shorter run-in period were used, then most men would appear in the earliest period, and the number of men with a history of repeated PSA measurements would be overrepresented. Using the 4-year cutoff makes the number of men included per day in each period similar.

These men were categorized according to screening and workup performed within 180 days after the first PSA testing, as follows: (1) no further action was taken (no action); (2) PSA was remeasured, but no MRI or no biopsy was performed (new PSA); (3) no MRI was performed, and the ensuing biopsy findings were negative (no MRI, negative biopsy); (4) MRI was performed, and biopsy findings were negative (MRI, negative biopsy); (5) MRI was performed, and no biopsy was performed (MRI, no biopsy); (6) MRI was performed, and the ensuing biopsy findings were positive (MRI, positive biopsy); (7) MRI was not performed, and the ensuing biopsy findings were positive (no MRI, positive biopsy); and (8) clinical diagnosis of prostate cancer was made with no MRI and no biopsy (clinical prostate cancer). This categorization was hierarchical, and the highest-ranking number (1-8) a man attained was registered as his outcome.

With this approach of 180 days to outcome, it was possible to reach an outcome for all men after their first PSA measurement. We found it reasonable that a PSA investigation in this setting was terminated after 180 days. If no time limit was set, the study would be hard to perform and interpret within the data set.

By use of the individually unique Swedish personal identity number, we linked men with a prostate cancer diagnosis in the study group to the NPCR of Sweden. In brief, NPCR contains information on the date of diagnosis, tumor stage, biopsy Gleason score, serum PSA level, mode of detection, and executed or planned primary treatment. Since 2007, prostate volume, total number of cores obtained at the diagnostic biopsy session, number of cores containing cancer, and extent of cancer in millimeters in all cores combined are registered.^9^ NPCR has been validated and showed that data quality in NPCR is high.^10^

**Figure 1.**
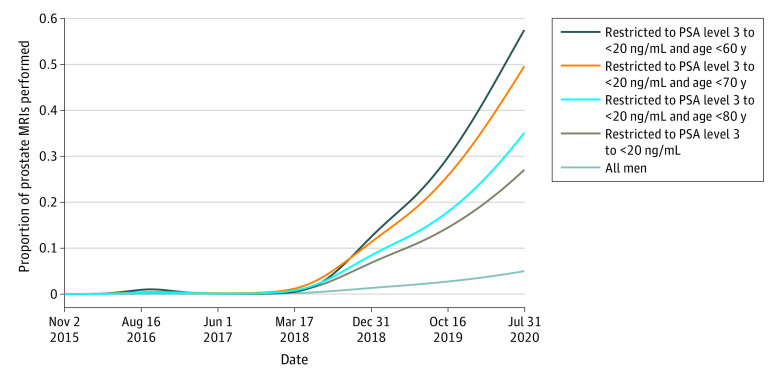
Use of Prostate Magnetic Resonance Imaging (MRI) Over Time Graph shows proportion of MRIs performed as a function of the first prostate-specific antigen (PSA) value on November 1, 2015, to July 31, 2020, for all men, and for men with PSA between 3 and less than 20 ng/mL according to age groups. To convert PSA to micrograms per liter, multiply by 1.

In the Swedish national guidelines for prostate cancer,^3^ MRI of the prostate before biopsy has been recommended since 2018. Because the implementation of MRI in Jönköping Region was slow in the beginning of the study period and then gradually caught up, we divided the study in 2 periods: the first was when the use of MRI was low (until February 28, 2018) and the second (from March 1, 2018) was when the use of MRI gradually became higher (Figure 1). In Jönköping Region, all MRI readings were collected at 1 center and all MRI-targeted prostate biopsies were performed there. All targeted biopsies were done with a bk3000 ultrasonography system with bkFusion, powered by MIM Software version 7.2.2.M127-01 (all from BK Medical). Fusion-guided biopsies have been reported to be superior to cognitively guided biopsies.^11^ All men with Prostate Imaging Reporting & Data System^12^ grade 3 to 5 lesions were recommended to undergo biopsy. If the PSA level was greater than 0.15 ng/mL (to convert PSA to micrograms per liter, multiply by 1), systematic biopsies were planned regardless of Prostate Imaging Reporting & Data System grade, according to the national guidelines.^13^

When MRI was introduced in Sweden, most radiologists involved in reading prostate MRIs in Jönköping Region participated in a national prostate MRI training course. In the beginning, MRI assessments were re-examined by an experienced external MRI radiologist, and consensus was reached. The biopsies were examined by the same uropathologists during the entire study period, and there were 4 trained urologists (D.R., R.A., D.N., and an individual who was not a coauthor of this article) who performed all biopsies.

### Statistical Analysis

Data analysis was performed from July to December 2022. The proportion of men undergoing MRI was modeled through a logistic regression model using natural cubic spline of calendar time as exposure. This was done in separate strata depending on age and PSA levels.

The distribution of groups 1 to 8 in relation to calendar time was calculated by splitting the follow-up time into 20 time slots of equal size. The proportions within each time slot were illustrated by use of locally weighted scatterplot smoothing estimates. This was done separately for men with a PSA level of 3 to less than 20 ng/mL and for the full cohort.

The approach of splitting the follow-up time was also used in an analysis of the outcome of the biopsies. Incidence of the different outcomes of the biopsies was calculated. The denominator for incidence was based on the person-years spent by the men in the county in each time slot, taking the increase in population size into account. Again, locally weighted scatterplot smoothing estimates were used to illustrate the proportions and incidences.

Logistic regression was used to assess the association of the exposure (low or high use of MRI) with outcomes in a univariable model and in an adjusted model adjusting for PSA categorized into 5 levels (0 to <3 ng/mL, 3 to <10 ng/mL, 10 to <20 ng/mL, 20 to <50 ng/mL, and ≥50 ng/mL). The outcome of our study was the proportion of Gleason score 6 and Gleason score 7 to 10 cancers, the performance of a biopsy (ie, a combination of groups 3, 4, 6, and 7), and performance of a negative biopsy (ie, a combination of groups 3 and 4). Finally, we calculated the outcome of the Gleason grading of the biopsies as incidence per 100 000 estimated total population. A sensitivity analysis was performed in which the use or nonuse of MRI for men with prostate cancer detected and initial PSA level of 3 ng/mL to less than 20 ng/mL and age younger than 80 years was performed. Data were analyzed using R statistical software version 4.1.2 (R Project for Statistical Computing).

## Results

**Figure 2.**
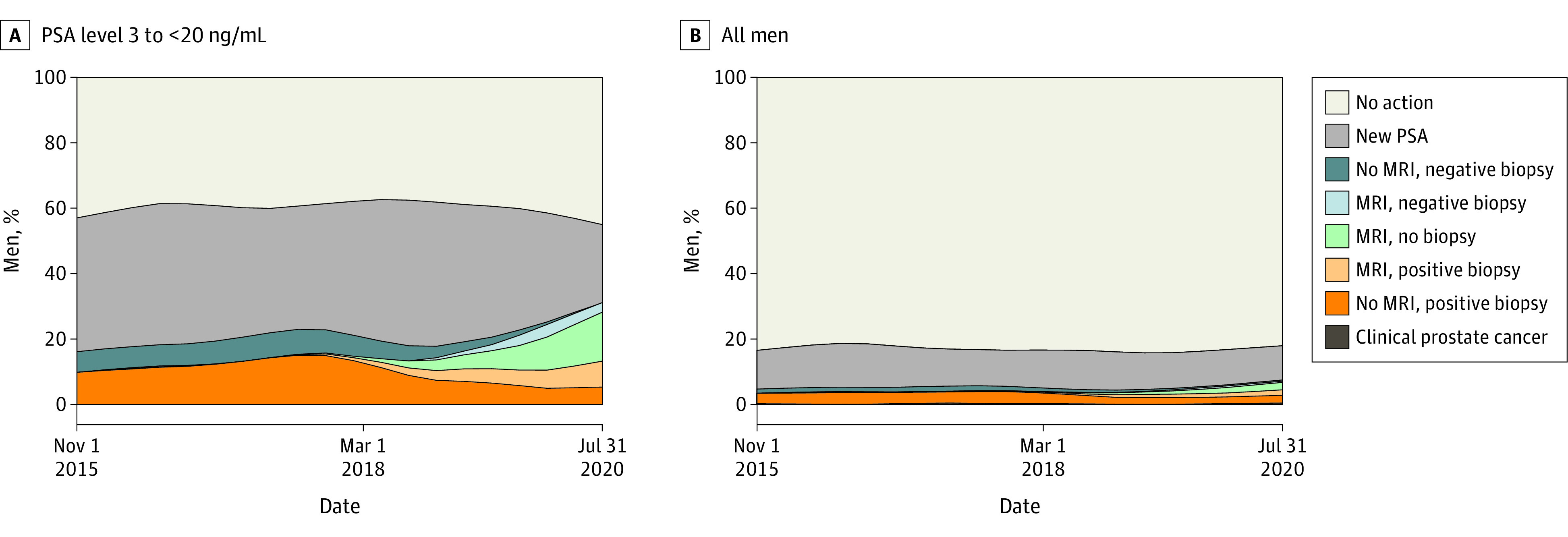
Workup Within 180 Days After the First Prostate-Specific Antigen (PSA) Test Among Men in Jönköping Region, Sweden, 2015-2020 Graphs show data for men with PSA level between 3 and less than 20 ng/mL (A) and men with any PSA level (B). This categorization was hierarchical, and a man could be registered with only 1 workup. To convert PSA to micrograms per liter, multiply by 1. MRI indicates magnetic resonance imaging.

**Figure 3.**
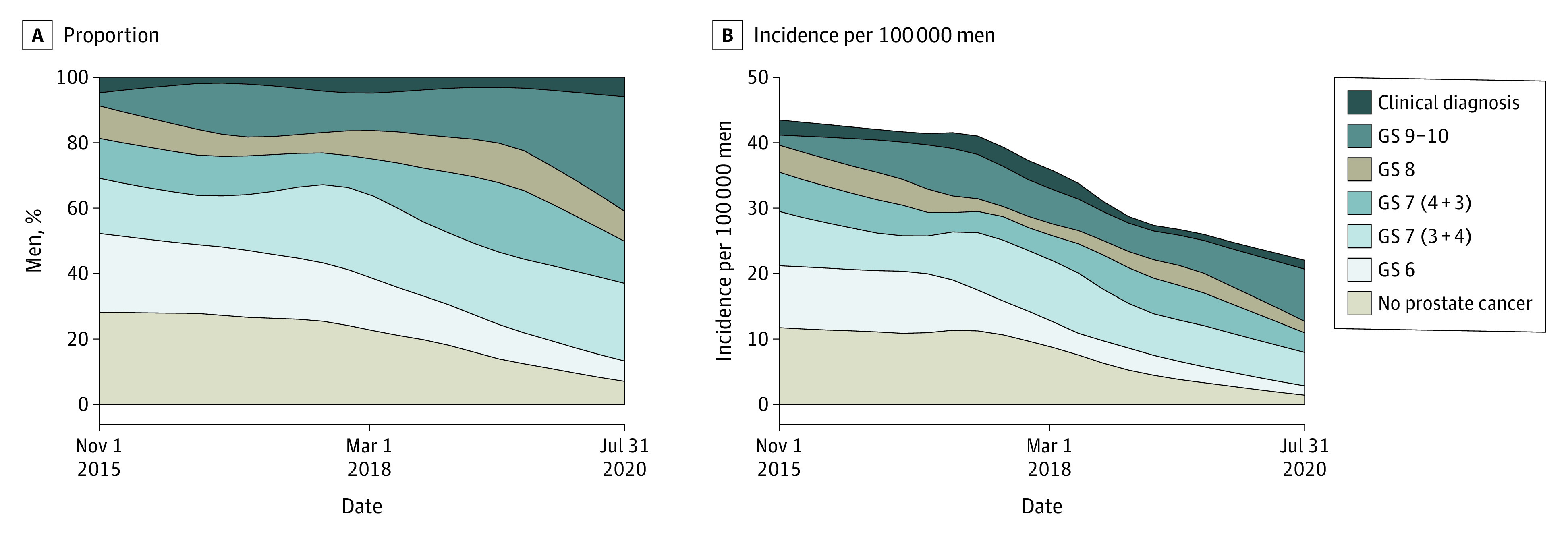
Outcomes of Biopsies Graphs show the outcome of the biopsies as proportions of men (A) and incidence per 100 000 estimated total population (B), according to Gleason score (GS). Clinical diagnosis means that no biopsy was performed, and the findings were benign.

In this cohort study of 23 802 men (mean [SD] age, 60.8 [13.6] years) who underwent PSA testing, the proportion of men with PSA greater than 20 ng/mL remained unchanged during the study period, and there was a slight increase of men with PSA less than 3 ng/mL and slightly fewer men with PSA of 3 to less than 10 ng/mL (Table 1). At the end of the study period, approximately one-half of the men with PSA between 3 and 20 ng/mL still had a diagnosis of prostate cancer without MRI being performed before diagnosis (Figure 2). There was a decrease in the proportion of men with negative biopsies from 28% to 7%, and a concomitant decrease in Gleason score 6 cancers from 24% to 6%, whereas the proportion of Gleason score 7 to 10 cancers increased from 49% to 86% (Figure 3).

**Table 1.** Age at Blood Draw and First Serum PSA Level for Men in Jönköping Region, Sweden, 2016-2020

Characteristic	Participants, No. (%)					
	2016 (n = 6330)^a^	2017 (n = 5161)	2018 (n = 5286)	2019 (n = 4910)	2020 (n = 2111)^a^	2016-2020 (n = 23 802)
Age range, y						
≤35	193 (3.0)	178 (3.4)	191 (3.6)	196 (4.0)	107 (5.1)	866 (3.6)
36-45	563 (8.9)	456 (8.8)	457 (8.6)	468 (9.5)	190 (9.0)	2134 (9.0)
46-50	714 (11.3)	603 (11.7)	560 (10.6)	518 (10.5)	235 (11.1)	2630 (11.0)
51-55	815 (12.9)	706 (13.7)	778 (14.7)	664 (13.5)	338 (16.0)	3301 (13.9)
56-60	882 (13.9)	719 (13.9)	694 (13.1)	662 (13.5)	274 (13.0)	3232 (13.6)
61-65	874 (13.8)	695 (13.5)	716 (13.5)	629 (12.8)	260 (12.3)	3174 (13.3)
66-70	867 (13.7)	643 (12.5)	639 (12.1)	580 (11.8)	220 (10.4)	2949 (12.4)
71-75	581 (9.2)	493 (9.6)	552 (10.4)	479 (9.8)	196 (9.3)	2301 (9.7)
76-80	386 (6.1)	283 (5.5)	365 (6.9)	346 (7.0)	148 (7.0)	1530 (6.4)
81-85	269 (4.2)	220 (4.3)	186 (3.5)	209 (4.3)	78 (3.7)	962 (4.0)
≥86	186 (2.9)	165 (3.2)	148 (2.8)	159 (3.2)	65 (3.1)	723 (3.0)
PSA level, ng/mL						
0 to <3	4976 (78.6)	4099 (79.4)	4327 (81.9)	4053 (82.5)	1704 (80.7)	19163 (80.5)
3 to <10	1026 (16.2)	801 (15.5)	729 (13.8)	634 (12.9)	303 (14.4)	3493 (14.7)
10 to <20	185 (2.9)	136 (2.6)	125 (2.4)	118 (2.4)	48 (2.3)	612 (2.6)
20 to <100	102 (1.6)	94 (1.8)	84 (1.6)	77 (1.6)	39 (1.8)	396 (1.7)
≥100	41 (0.6)	31 (0.6)	21 (0.4)	28 (0.6)	17 (0.8)	138 (0.6)

Abbreviation: PSA, prostate-specific antigen.

SI conversion factor: To convert PSA to micrograms per liter, multiply by 1.

^a^ Data for 2016 correspond to PSA test performed from November 1, 2015, to December 31, 2016. Data for 2020 correspond to tests performed from January 1, 2020, to July 31, 2020.

We investigated time trends of use of MRI by use multivariable regression models. In the period when use of MRI had become high, fewer biopsies were performed compared with the earlier period (odds ratio [OR], 0.84; 95% CI, 0.72-0.97), and there was a decrease in risk of negative biopsies (OR, 0.70; 95% CI, 0.54-0.90) (Table 2). There was a decreased risk of Gleason score 6 cancer (OR, 0.47; 95% CI, 0.33-0.64), whereas the risk of Gleason score 7 or higher cancer increased (OR, 1.24; 95% CI, 1.02-1.50). The sensitivity analysis of men with prostate diagnosis and initial PSA of 3 to less than 20 ng/mL showed a reduced proportion of men with a Gleason score 6 cancer detected (16% vs 38%), with a corresponding increase in men with Gleason score 7 to 10 cancers (eTable in Supplement 1). The data were validated; PSA levels from health care records were compared with the PSA level recorded in NPCR, and 815 of 841 men (97%) had the same PSA level registered in both sources.

**Table 2.** Performance of Biopsy and Detection of Cancer on Biopsy According to Low or High Use of MRI^a^

Assessment and period^b^	No. of events/No. of PSA measurements (%)	OR (95% CI)	
		Crude	Adjusted^c^
Biopsy performed			
Early	656/11 952 (5.2)	1.00 [Reference]	1.00 [Reference]
Late	457/10 737 (4.1)	0.78 (0.69-0.88)	0.84 (0.72-0.97)
No cancer on biopsy			
Early	182/12 426 (1.4)	1.00 [Reference]	1.00 [Reference]
Late	100/11 094 (0.9)	0.62 (0.48-0.78)	0.70 (0.54-0.90)
Gleason score 6			
Early	135/12 473 (1.1)	1.00 [Reference]	1.00 [Reference]
Late	50/11 144 (0.4)	0.41 (0.30-0.57)	0.47 (0.33-0.64)
Gleason score 7-10			
Early	339/12 269 (2.7)	1.00 [Reference]	1.00 [Reference]
Late	307/10 887 (2.7)	1.02 (0.87-1.19)	1.24 (1.02-1.50)

Abbreviations: MRI, magnetic resonance imaging; OR, odds ratio; PSA, prostate-specific antigen.

^a^ Logistic regression was used to analyze exposure to low or high use of MRI and biopsy performed (yes or no) and outcome of biopsy Gleason grading of biopsies in a crude model and an adjusted model.

^b^ The early period refers to November 1, 2015, to February 28, 2018, when use of MRI was low. The late period refers to March 1, 2018, to July 31, 2020, when use of MRI had increased.

^c^ ORs were adjusted for PSA categorized into 5 levels (0 to <3, 3 to <10, 10 to <20, 20 to <50, and ≥50 ng/mL). To convert PSA to micrograms per liter, multiply by 1.

## Discussion

In this population-based cohort study, the introduction of MRI was associated with a decrease in prostate biopsies, a decrease in negative biopsies, and a decrease in the detection of Gleason score 6 cancer, whereas the detection of Gleason score 7 or higher cancer increased. These results are in accordance with the results from some previous RCTs^1,2^ and indicate that the advantages of MRI before prostate biopsy can also be achieved in clinical practice.

Several RCTs compared the use of MRI-targeted biopsies with systematic biopsies and their associations with biopsy frequency and Gleason score. In the PROMIS study,^5^ 27% of men had no findings on MRI, and biopsy could be omitted. In the MRI group, 18% more clinically important cancers (defined as a finding of Gleason score 4 + 3 or greater, or a maximum cancer involvement of ≥6 mm) were detected and 5% fewer clinically unimportant cancers were diagnosed. In the PRECISION study,^4^ 500 men were randomized to MRI-targeted biopsy or systematic biopsy. In the MRI group, 28% of the men had a normal MRI and did not undergo biopsy. Clinically important cancer (Gleason score 3 + 4 or greater) was detected in 38% of the men in the MRI group compared with 26% in the systematic biopsy group. Significantly fewer men in the MRI-targeted biopsy group than in the standard biopsy group received a diagnosis of clinically unimportant cancer (adjusted difference, −13%; 95% CI, −19% to −7%; *P* < .001).^4^

However, when men in a screening setting were randomized to MRI first or systematic biopsy, there was no significant difference in terms of proportion of men with clinically important cancer (Gleason score ≥7) between the 2 groups.^14^ Clinically important cancer was diagnosed in 21% of men in the MRI group, and 18% in the systematic biopsy group. The risk of clinically unimportant cancer was lower after MRI compared with systematic biopsy (4% vs 12%).^14^

In the recent Göteborg 2 prostate cancer screening study,^6^ the risk of Gleason score 6 cancer was 54% lower for men who underwent cognitive MRI-targeted biopsy compared with men who underwent systematic biopsy. However, omitting systematic biopsies resulted in a decreased detection of Gleason score 7 or higher cancer by 19%.

In these 4 RCTs,^4,5,6,14^ MRI first vs systematic biopsy detected fewer clinically unimportant cancers, and for men with a clinical suspicion of prostate cancer, more clinically important cancers were detected.^4,5^ However, for men in a screening setting, there was no difference or even a decreased incidence of clinically important prostate cancer.^6,14^

### Limitations and Strengths

There are some limitations to this study. First, the number of men was too small to study some pertinent subgroups (eg, men who had undergone a previous biopsy) or to analyze the use of MRI according to PSA velocity. The study period included the peak of the COVID-19 pandemic, during which there was a decrease in PSA testing.^15^ However, the distribution of PSA levels in our study was unchanged compared with the period before the pandemic, and it is unlikely that this factor would be a confounder influencing our results in a favorable way for the MRI-first path. Even during the pandemic, the use of MRI increased. Another limitation to this study was that at the time of the study, the targeted and systematic biopsies from the prostate were not clearly separated when they were reported in the NPCR. Our assessment of cancer aggressiveness by dichotomizing the Gleason score as 6 vs 7 to 10 is a simplification, since the goal is to find men who will die from prostate cancer; however, it has been used in several previous studies.^4,5,6,14^

This study has several strengths. First, it is truly population-based since there were few men who received health care outside the tax-funded system in Jönköping Region, Sweden (ie, there was strong internal validity). We argue that the results could be replicated elsewhere (external validity) if guidelines are followed and the urologist and MRI radiologist are dedicated. To the best of our knowledge, this is the first study on the effect of the introduction of MRI-targeted biopsies on biopsy frequency and biopsy grading in clinical practice. In addition to data collected from health care records, we used data from the NPCR, a clinical cancer register with documented high capture and data quality.^10^

## Conclusions

In this population-based cohort study, the introduction of MRI to clinical practice before making a decision on prostate biopsy was associated with decreased numbers of performed biopsies, decreased numbers of negative biopsies, and decreased numbers of Gleason score 6 cancers detected, whereas the detection of Gleason score 7 or higher cancer increased. Our results are in line with those of previous RCTs of men with clinical suspicion of prostate cancer. Our results support that all men with PSA between 3 and 20 ng/mL should undergo MRI before biopsy. These results need to be replicated and expanded in larger cohorts in clinical practice to study pertinent subgroups.

## Notes

Supplement 1. **eFigure.** Consort Diagram of Men Included in the Study **eTable.** Distribution of Prostate Cancer Characteristics by MRI Investigation for Men Diagnosed With Needle Biopsy Detected Prostate Cancer With First PSA Between 3 and 20 ng/ml and Age Below 80 Years

Supplement 2.
Data Sharing Statement
