# Supplementary material for: Frequency of Biopsy and Tumor Grade Before vs After Introduction of Prostate Magnetic Resonance Imaging
Source: JAMA Netw Open. 2023 Aug 22;6(8):e2330233. doi: 10.1001/jamanetworkopen.2023.30233 (PMC10445184; doi:10.1001/jamanetworkopen.2023.30233)
Supplement: Supplement 1. — eFigure. Consort Diagram of Men Included in the Study eTable. Distribution of Prostate Cancer Characteristics by MRI Investigation for Men Diagnosed With Needle Biopsy Detected Prostate Cancer With First PSA Between 3 and 20 ng/ml and Age Below 80 Years [file jamanetwopen-e2330233-s001.pdf]

Supplemental Online Content

Robinson D, Abdulkareem R, Nasrollah D, et al. Frequency of biopsy and tumor grade before vs after introduction of prostate magnetic resonance imaging. *JAMA Netw Open*. 2023;6(8):e2330233. doi:10.1001/jamanetworkopen.2023.30233

eFigure. Consort Diagram of Men Included in the Study

eTable. Distribution of Prostate Cancer Characteristics by MRI Investigation for Men Diagnosed With Needle Biopsy Detected Prostate Cancer With First PSA Between 3 and 20 ng/ml and Age Below 80 Years

This supplemental material has been provided by the authors to give readers additional information about their work.

© 2023 Robinson D et al. *JAMA Network Open.*

eFigure. Consort Diagram of Men Included in the Study.

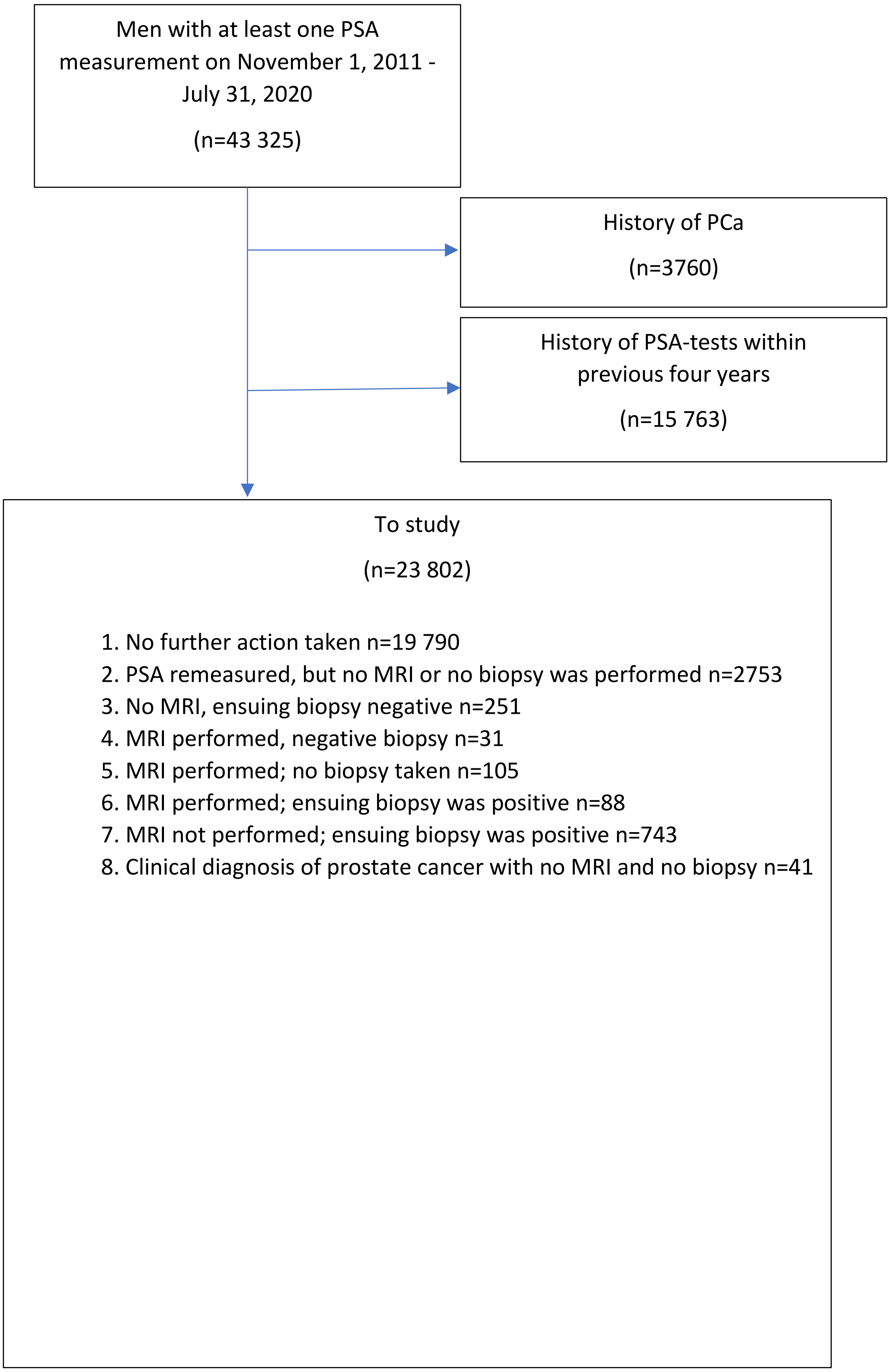

© 2023 Robinson D et al. *JAMA Network Open.*

eTable. Distribution of Prostate Cancer Characteristics by MRI Investigation for Men Diagnosed With Needle Biopsy Detected Prostate Cancer With First PSA Between 3 and 20 ng/ml and Age Below 80 Years.

	MRI (n=67)		No MRI (n=363)		Total (n=430)	
**Age, years n (%)**						
<=55	9	(13.4)	36	(9.9)	45	(10.5)
56-66	27	(40.3)	126	(34.7)	153	(35.6)
66-75	30	(44.8)	158	(43.5)	188	(43.7)
76-80	1	(1.5)	43	(11.8)	44	(10.2)
**T-stage, n (%)**						
T1ab	1	(1.5)	4	(1.1)	5	(1.2)
T1c	36	(53.7)	177	(48.8)	213	(49.5)
T2	25	(37.3)	152	(41.9)	177	(41.2)
T3	5	(7.5)	24	(6.6)	29	(6.7)
T4	0	(0.0)	4	(1.1)	4	(0.9)
TX	0	(0.0)	2	(0.6)	2	(0.5)
**PSA-density, n (%)**						
0-0.15	20	(29.9)	112	(30.9)	132	(30.7)
0.15-0.30	29	(43.3)	153	(42.1)	182	(42.3)
0.30+	18	(26.9)	90	(24.8)	108	(25.1)
Missing	0	(0.0)	8	(2.2)	8	(1.9)
**Gleason, n (%)**						
6	11	(16.4)	137	(37.7)	148	(34.4)
7(3+4)	30	(44.8)	135	(37.2)	165	(38.4)
7(4+3)	15	(22.4)	48	(13.2)	63	(14.7)
8	5	(7.5)	21	(5.8)	26	(6.0)
9-10	6	(9.0)	22	(6.1)	28	(6.5)
**Treatment, n (%)**						
Active surveillance	8	(11.9)	88	(24.2)	96	(22.3)
Watchful waiting	1	(1.5)	26	(7.2)	27	(6.3)
Radical prostatectomy	33	(49.3)	132	(36.4)	165	(38.4)
Radiation therapy	19	(28.4)	81	(22.3)	100	(23.3)
Androgen deprivation therapy	6	(9.0)	29	(8.0)	35	(8.1)
Missing information	0	(0.0)	7	(1.9)	7	(1.6)

© 2023 Robinson D et al. *JAMA Network Open.*
