# Supplementary material for: Frequency of Biopsy and Tumor Grade Before vs After Introduction of Prostate Magnetic Resonance Imaging
Source: JAMA Netw Open. 2023 Aug 22;6(8):e2330233. doi: 10.1001/jamanetworkopen.2023.30233 (PMC10445184; doi:10.1001/jamanetworkopen.2023.30233)
Supplement: Supplement 2. — Data Sharing Statement [file jamanetwopen-e2330233-s002.pdf]

Data Sharing Statement

Robinson. Frequency of Biopsy and Tumor Grade Before vs After Introduction of Prostate Magnetic Resonance Imaging. *JAMA Netw Open*. Published August 22, 2023. doi:10.1001/jamanetworkopen.2023.30233

Data
Data available: No
